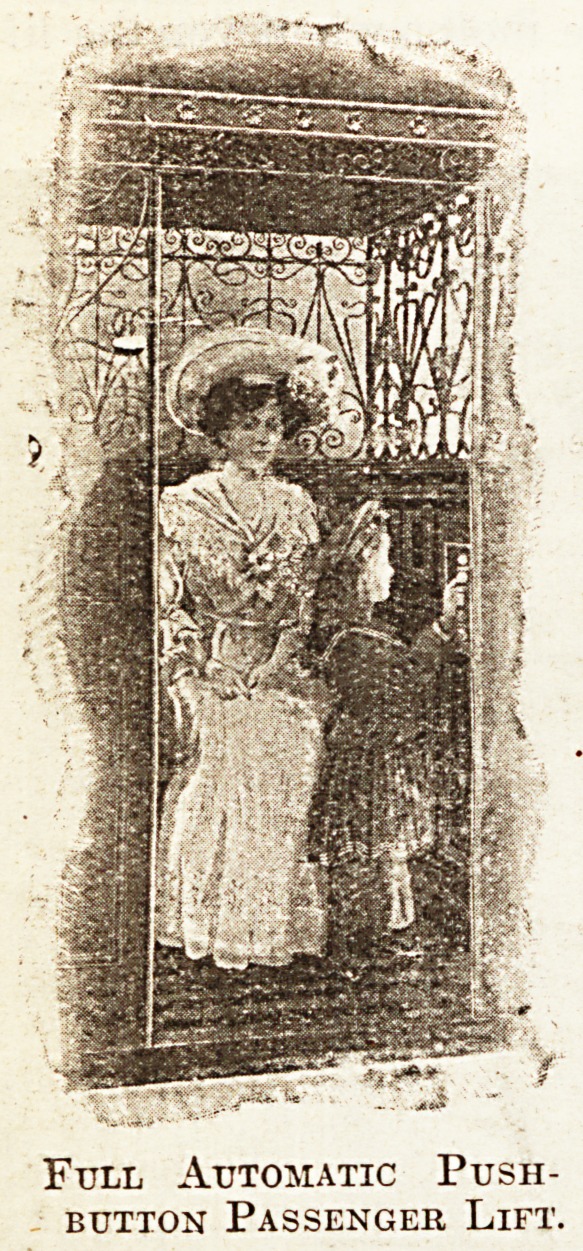# The Push-Button Automatic Lift

**Published:** 1920-02-21

**Authors:** 


### ELECTRIC LIFTS FOR HOSPITALS.

### The Push-Button Automatic Lift.

THE lift has long been a necessity of all hospital buildings. For a number of years the hydraulic lift did good service, but it has gradually been displaced by the electric lift. The lift problem is only one of many in which electricity is superseding the older agents employed, principally because of the great convenience with which it can be worked, and the greater number of arrangements that can be made with it. The electric lift has been in use for a number of years worked from the cage, but it is being gradually displaced by the "push-button automatic lift." The drain upon the staffs of a great many hospitals has obliged labour to be saved wherever it is practicable, and the push-button automatic lift saves an attendant.

HAND-OPERATED HOSPITAL LIFT.

### THE ARRANGEMENT OF AN ELECTRIC LIFT.

In order that the working of the push-button automatic lift may be grasped, it will perhaps be as well to give a short description of the working arrangements of all electric lifts, and then to explain the special apparatus enabling the automatic system to be worked. The lift itself, it will be remembered, is a platform with sides and a roof forming a small chamber. The chamber, or cage, is suspended in the shaft provided for it by steel wire ropes passing over pulleys at the top of the shaft and attached to the roof of the cage. The cage moves in guides in the shaft, and a balance weight, carried by wire ropes passing over the pulleys at the top of the shaft, moves up and down as the cage moves down and up. The end of the rope that supports the cage is attached to an iron drum, sometimes placed in the basement, and sometimes at the top of the shaft. The iron drum is made to revolve by an electric motor, whose axle is connected to that of the drum. The motor revolves in either direction at will, winding up the rope of the drum when turning in one direction and thus raising the cage in the shaft, and allowing the rope to unwind from the drum when it revolves in the opposite direction, so that the cage descends. The current for the motor may be supplied from the town service, through the usual entry switch and meter, with a separate switch to cut off the current from the lift entirely when required, or it may be worked by current generated in the hospital. The important point is that current must be available for driving the motor whenever the lift is required to be used, and it must be possible to cut off completely all the lift apparatus from the electric supply for overhaul of the arrangements.

ELECTRICALLY-DRIVEN BED LIFT FOR HOSPITALS.

### STARTING THE LIFT.

With all electric motors, certain arrangements are necessary when starting. The current must not be allowed to pass into the motor at full pressure when the motor is at rest. So that what are called motor starters are attached to all electric motors. The motor starter is arranged first to energise the magnetic field of the motor, and then to apply a gradually increasing pressure to the coils of the motor armature as the speed gets up. In the electric lift these operations are performed automatically whenever the lift is started, by the aid

**Electric Lifts for Hospitals**—*(continued)*.

of a series of electro-magnets. What the attendant of an electric motor does by moving a handle along a graduated surface, the electro-magnets fixed upon a switchboard in the neighbourhood of the lift motor perform automatically. First one electro-magnet closes the circuit of the field magnets of the motor and produces a powerful magnetic field, then other electro-magnets, one after another in succession, first connect the armature of the motor to the electric service through a graduated resistance, and then gradually cut the resistance out until the full pressure of service is operating in the motor coils and the motor revolves at its full speed, and the cage moves up or down at the speed for which it was designed. In addition to this the motor, and with it the drum, is kept at rest when the cage is not moving, by a powerful brake applied to a pulley fixed for the purpose upon the axle of the drum, upon which the rope is coiled. When the lift is to start, in addition to the field magnets of the motor being energised, a powerful electro-magnet pulls off the brake and sets the drum free to revolve. A special arrangement of the coils upon the field magnet is also used with electric lifts to give a very strong starting effort: what engineers called a powerful torque.

FULL AUTOMATIC PUSH-BUTTON PASSENGER LIFT.

THE PUSH-BUTTON ARRANGEMENT.

With the push-button automatic lift there is a push outside the shaft on each landing, and there are usually pushes on the cage itself. Each push merely completes a circuit in which the starting apparatus of the motor is included. An electro-magnet in the circuit of each push operates a lever which causes the magnetic field of the motor to be energised, the brake to be released, and the current to be allowed to pass into the armature coils of the motor. There is an electro-magnet at the switchboard near the motor for each push; each electro-magnet closes a circuit which performs all the offices mentioned above. But the push-button control, in addition to causing the lift to move up or down automatically, also causes it to stop at the landing where the push has been pressed. The pushes on the lift itself are arranged to cause the lift to move upwards, downwards, or to come to rest. Their principal use is when the shaft is being overhauled from the cage. They are also of use to bring the cage back to a floor when someone wishes to board the cage after it has left that floor.

In addition to the other apparatus mentioned, there is another series of electric switches, put in operation by arms attached to a revolving axle fixed in the neighbourhood of the switchboard near the motor. The axle is caused to revolve by a chain passing over a pulley on the end of the axle of the motor, and another pulley on an extension of the axle of the motor. The arms carried by the axle are placed at different angles, the angles being arranged so that each arm comes to its contact exactly at the time required, and when the cage has moved up or down through the distance required of it. When the cage is called to the first floor, say from the fourth floor, the motor revolves, unwinding the rope and causing the cage to descend to the first floor, and the arm belonging to that push moves round until, when the rope has run out to the extent required for the cage to descend to the first floor, the arm corresponding opens a switch in the circuit, causes the current to cease to flow through the motor, and at the same time the brake comes into operation and the motor and the cage come to rest. The arrangements for stopping the lift from a push on the cage and for causing the cage to move up and down are worked in a similar manner.

SAFETY ARRANGEMENTS.

One important feature claimed for electric lifts is their safety. With the push-button automatic lift it is not possible to set the lift in motion if the collapsible gates opening on to the landings are open. There is a collapsible gate to the cage itself and a second one on the landing, and both must be closed on all the floors before the lift will move. This has one drawback. If anyone, after using the lift, neglects to shut the gates, no one can use the lift from the other floors. In addition, in case of the rope breaking or the lift overrunning, or of its descending too sharply to the bottom, the current is immediately cut off and the brake comes into operation, causing the motor to come to rest and the cage with it. The whole of the safety appliances are arranged on a very simple system. All the gates have automatic switches, which are closed when the gates are closed, and are open when the gates are open. The cage switches and safety switches at top and bottom of the shaft form part of the motor circuit. If any one of the switches is open no current can pass through the motor, and the brake which prevents the motor and drum revolving remains in position; also the brake comes into operation and the current is cut off from the motor immediately in case of any accident anywhere.

## Figures and Tables

**Figure f1:**
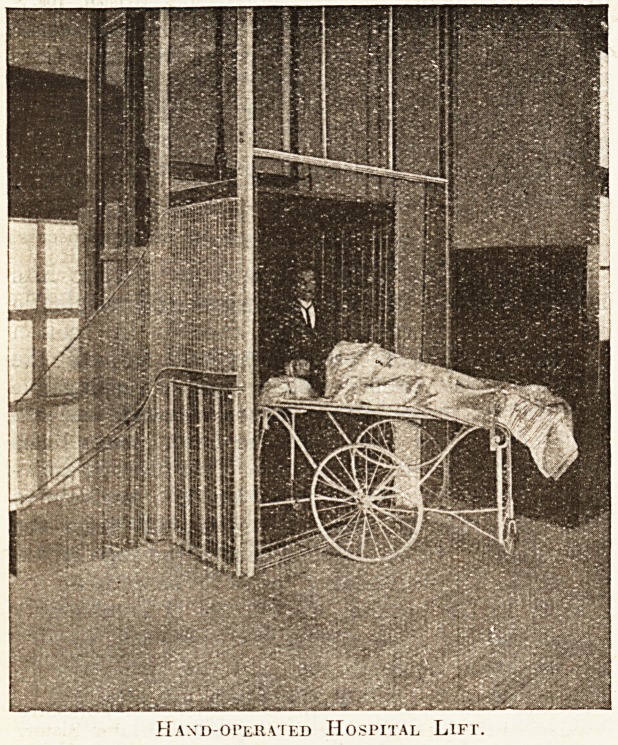


**Figure f2:**
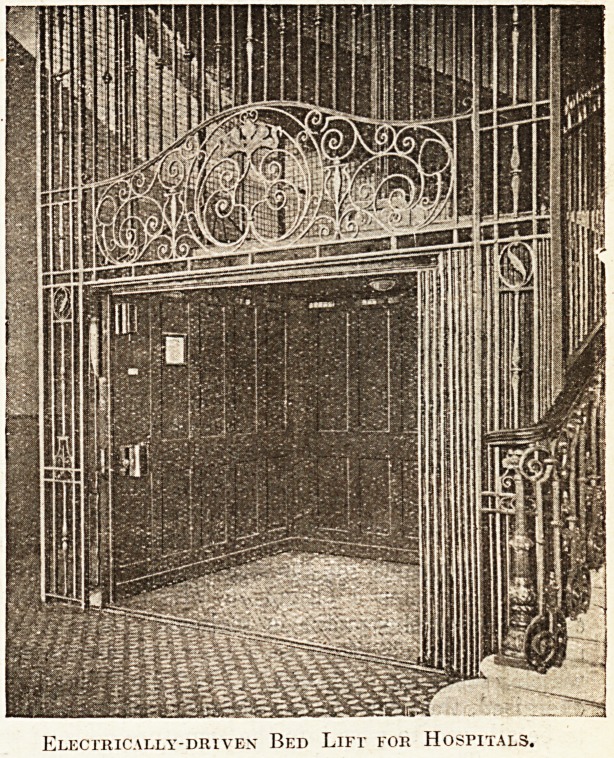


**Figure f3:**